# *Verticillium longisporum* Infection Affects the Leaf Apoplastic Proteome, Metabolome, and Cell Wall Properties in *Arabidopsis thaliana*

**DOI:** 10.1371/journal.pone.0031435

**Published:** 2012-02-20

**Authors:** Saskia Floerl, Andrzej Majcherczyk, Mareike Possienke, Kirstin Feussner, Hella Tappe, Christiane Gatz, Ivo Feussner, Ursula Kües, Andrea Polle

**Affiliations:** 1 Department of Forest Botany and Tree Physiology, Büsgen-Institute, Georg August University, Göttingen, Germany; 2 Department of Molecular Wood Biotechnology and Technical Mycology, Büsgen-Institute, Georg August University, Göttingen, Germany; 3 Department of Plant Biochemistry, Albrecht von Haller Institute, Georg August University, Göttingen, Germany; 4 Department of Molecular Biology and Physiology of Plants, Albrecht von Haller Institute, Georg August University, Göttingen, Germany; Friedrich-Alexander-University Erlangen-Nurenberg, Germany

## Abstract

*Verticillium longisporum* (VL) is one of the most devastating diseases in important oil crops from the family of Brassicaceae. The fungus resides for much time of its life cycle in the extracellular fluid of the vascular system, where it cannot be controlled by conventional fungicides. To obtain insights into the biology of VL-plant interaction in the apoplast, the secretome consisting of the extracellular proteome and metabolome as well as cell wall properties were studied in the model Brassicaceae, *Arabidopsis thaliana*. VL infection resulted in increased production of cell wall material with an altered composition of carbohydrate polymers and increased lignification. The abundance of several hundred soluble metabolites changed in the apoplast of VL-infected plants including signalling and defence compounds such as glycosides of salicylic acid, lignans and dihydroxybenzoic acid as well as oxylipins. The extracellular proteome of healthy leaves was enriched in antifungal proteins. VL caused specific increases in six apoplast proteins (three peroxidases PRX52, PRX34, P37, serine carboxypeptidase SCPL20, α-galactosidase AGAL2 and a germin-like protein GLP3), which have functions in defence and cell wall modification. The abundance of a lectin-like, chitin-inducible protein (CILLP) was reduced. Since the transcript levels of most of the induced proteins were not elevated until late infection time points (>20 dpi), whereas those of *CILLP* and *GLP3* were reduced at earlier time points, our results may suggest that VL enhances its virulence by rapid down-regulation and delay of induction of plant defence genes.

## Introduction

*Verticillium* species are wide-spread soil borne fungi, which cause vascular diseases in many plant species [1]. Most studies addressed plant responses to *V. dahliae* or *V. albo-atrum*, which have the largest host range and cause billions of dollars of yield loss in crops worldwide [2]. In addition, *V. longisporum* (VL) infection has been identified in the last decade as one of the most important diseases of Brassicaceae, in particular of oilseed rape [3], [4]. To date, the economic importance and acreage of oilseed rape are increasing because of growing demand on oil crops for nutrition and bio-fuels [2], [5], [6]. This is accompanied by a spread of VL diseases, which may cause yield losses as high as 10 to 50% [7]. Since diseases caused by *Verticillium* spp. cannot be controlled by conventional fungicides, the use of resistant cultivars has been recommended [7]. Breeding of resistant cultivars requires in-depth understanding of the biology of the VL-host interaction.

*Verticillium* species infect their hosts by root penetration and subsequently colonize the xylem [1], where they cause partial clogging of the vessels [8]. These obstructions are expected to affect water and nutrient transport. Therefore, typical disease symptoms such as wilting, stunting, chlorosis and premature senescence have been suggested to occur as consequences of water limitations and insufficient nutrient supply [9]. In contrast to other target plants, wilting symptoms were not observed in oilseed rape or in *A. thaliana* infected with VL [10]–[12]. Analyses of the plants' nutrient status during the VL infection cycle did not reveal nutrient limitations in these plant species [10], [11]. However, the VL-infected plants exhibited severe stunting indicating that the plant-pathogen interaction resulted in extensive re-modelling of plant architecture [10], [11], [13].

Since VL colonizes the plant by extracellular growth in the xylem, its influence on the apoplast is of particular interest. The apoplast is the space outside the plasma membrane in which water, nutrients, solutes and signals are exchanged between plant cells and invading organisms. It is the first compartment where contact between pathogen and plant is established and where primary defences are activated. The metabolism in this compartment is complex since it serves transport, sensing, defence as well as construction and maintenance of cell walls whose composition is tissue-specific and flexibly adjustable, for example when construction of barriers is required to prevent spread of invading pathogens [14]–[16]. This complexity is reflected by the existence of a huge number of secretory proteins, which have been estimated to account for about 17% of all genes in the *A. thaliana* genome [17]. The functions of most of these proteins are still unknown.

Preceding studies have shown atypical disparity between symptom development and pathogen proliferation indicating unusual features of *Verticillium*-host plant interactions [18]. In tomato a resistance locus Ve was identified to encode a cell surface glycoprotein with a leucine rich receptor domain [19], [20]. Over-expression of this gene in susceptible potato conferred tolerance to *V. dahliae* [19]. Comparison of nearly isogenic lines of *ve/ve* and *Ve/Ve* tomatoes showed that resistant cultivars showed stronger peroxidase activation, increased H_2_O_2_ and lignin formation in roots leading to stronger fungal attenuation than in the susceptible cultivar [21]. This suggests that apoplastic proteins play crucial roles in perception and activation of defences against the spread of *Verticillium* diseases.

Since information on the secretome of *Arabidopsis thaliana* under the influence of *V. longisporum* is lacking, the goal of this study was to characterize changes in the apoplast proteome and metabolome of leaves in relation to disease symptoms and cell wall properties. Transcriptional patterns of VL-responsive proteins were analyzed to investigate the time course of VL-induced regulation. Cell wall production and lignification increased in response to VL and cell wall carbohydrate composition was changed providing insights into dual functions of the identified proteins in basal defence and cell wall metabolism.

## Results

### *V. longisporum* infection results in foliar disease symptoms, but not in loss of membrane integrity

About three weeks after root infection with VL, *Arabidopsis* plants showed stunting (reduction in projected leaf area −57%) and reductions in rosette biomass (−40%), but only moderate chlorosis (chlorophyll −11%, carotenoids −10%) compared with mock-infected controls (Table 1). The electrolyte leakage, which is an indicator of membrane integrity, was low and not affected by VL (Table 1).

**Table 1:** *Verticillium longisporum* VL43 induced disease symptoms in *Arabidopsis thaliana*.

Parameter	n	Mock	VL43	*p*
Rosette area (mm^2^ plant^−1^)	30	6402±276	2750±181	0.0001
Fresh mass (g plant^−1^)	15	1.09±0.08	0.66±0.09	0.0018
Chlorophyll (µmol g^−1^ FW)	18	1135±21	1005±28	0.0008
Carotenoids (µmol g^−1^ FW)	18	271±6	245±6	0.0058
Electrolyte leakage (%)	5	15.5±0.9	16.2±0.9	0.6367
Lignin (mg g^−1^ DW)	8	39.4±1.5	48.6±2.6	0.0039

Measurements were taken 24 or 25 days post inoculation on the indicated number (n) of plants. Data indicate means (± SE). Values of *p*≤0.05 indicate significant differences between mock-inoculated and *Verticillium longisporum* (VL)-infected plants.

### The *Arabidopsis* apoplastic leaf proteome is affected by *V. longisporum*

To investigate the apoplastic proteome, we extracted apoplastic washing fluids (AWFs) from mock- and VL-infected rosette leaves of *Arabidopsis* plants at 25 dpi. Since leakage of symplastic components into the apoplast cannot be completely avoided during the extraction procedure, control of this contamination is essential. Determination of malate dehydrogenase (MDH) activity as a cytosolic marker enzyme showed that leakage was low and not influenced by VL infection (Table 2).

**Table 2:** Purity of apoplastic washing fluid (AWF) from leaves of mock- and *Verticillium longisporum* VL43-infected leaves.

Treatment	MDH (nkat g^−1^ FW)		Contamination
	Whole leaf	AWF	%
Mock	242.1±29.9	0.007±0.013	0.0030
VL43	220.6±59.7	0.007±0.004	0.0032

Plants were harvested 25 days post infection and foliage of 10 plants was pooled to obtain AWF. Five independent pools were analyzed per treatment. Data indicate means (± SE) for malate dehydrogenase activity, which is a symplastic marker.

**Figure 1:**
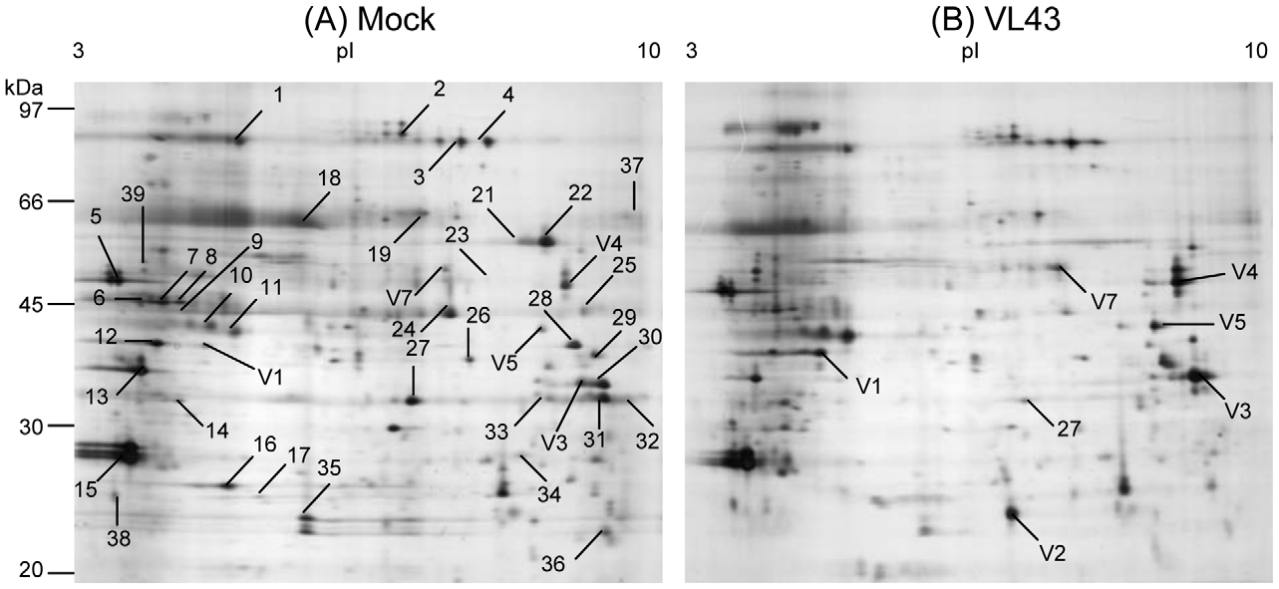
Typical 2-D gels showing *Arabidopsis thaliana* apoplastic leaf proteins after mock (A) or *Verticillium longisporum* **(VL43) infection (B).** Proteins were extracted 25 dpi and 80 µg were loaded for separation. Gels were stained with silver nitrate. Numbers in the gels indicate protein spots analyzed by ESI-LC/MS from preparative Coomassie-stained gels.

The protein content did not differ in leaf extracts or in AWFs of infected compared with non-infected plants (Table 3). 2-D-electrophoretic separation and silver staining of apoplastic proteins revealed mean spot numbers of 217 (±7) for AWFs from both mock- and VL-infected plants with no significant differences between treatments (Table 3). New spots were not detected. Coomassie-stained gels were used to observe and pick clearly discernable spots for mass spectrometry. Forty-five protein spots were identified (Fig. 1A, B). Statistical analysis revealed that thirty eight of these spots were unaffected and seven showed significant changes in response to VL infection (Table 4, Table 5, Table S1). Fungal proteins were not identified. Thirty-six of the identified proteins were predicted to have secretory signal peptides and were therefore considered as apoplastic proteins; one of them has no known function but was previously found in cell wall extracts of *Arabidopsis* [22]; three were predicted to be located in mitochondria and seven in chloroplasts.

**Table 3:** Influence of *Verticillium longisporum* VL43 infection on protein content of whole leaf extracts, in apoplastic washing fluid and on the number of protein spots after 2-D electrophoresis.

Parameter	N	Mock	VL43	*p*
Leaf protein (mg g^−1^ f.wt)	5	12.5±1.2	12.3±0.5	0.9192
AWF protein (µg g^−1^ f.wt)	5	17.2±1.0	21.0±2.4	0.1785
Protein spots (gel^−1^)	6	214±10	220±11	0.7389

Plants were analyzed 25 days post infection. Protein spots were determined by Proteomweaver software in silver stained gels (cf. Fig. 1). Data indicate means (N ± SE).

**Table 4:** Proteins identified by LC-MS/MS in the apoplast of *Arabidopsis thaliana* leaves.

Spot No	Similarity to	Locus	Proteinscore	expectedMw (Da)	Signal peptide
1	Thioglucosidase	At5g26000	123	61664	S (RC 1)
2	Subtilase	At5g67360	301	80050	S (RC 1)
3	Subtilase	At5g67360	241	80050	S (RC 1)
4	β-1,4-xylosidase	At3g19620	243	97359	S (RC 1)
5	Aspartyl protease	At5g10760	102	50411	S (RC 2)
6	Peptidyl-prolyl cis-trans isomerase	At3g01480	62	48180	C (RC 1)
7	Pepsin A	At3g18490	62	53942	C (RC 5)
8	Pepsin A	At3g18500	63	53942	M (RC 4)
9	Sedoheptulose-bisphosphatase precursor	At3g55800	168	42787	C (RC 1)
10	Curculin-like	At1g78830	169	51007	S (RC 1)
11	Curculin-like	At1g78830	203	51007	S (RC 1)
12	Carboxylic ester hydrolase	At1g29660	270	40630	S (RC 2)
13	β-1,3-glucanase 2 (PR2)	At3g57260	389	37373	S (RC 1)
14	Oxygen-evolving enhancer protein (OEE33)	At5g66570	66	35285	C (RC 2)
15	PR5, thaumatin-like	At1g75040	576	26148	S (RC 1)
16	Chaperonin 10	At1g14980	101	26913	M (RC 3)
17	Oxygen-evolving enhancer protein 2 (PSBP)	At1g06680	136	28249	C (RC 2)
18	Rubisco (large subunit)	AtCg00490	113	53424	_ (RC2)*
19	Serine hydroxyl-methyltransferase (SHM 1)	At4g37930	44	57535	M (RC 4)
21	Pepsin A, aspartyl protease	At1g09750	39	48429	S (RC 1)
22	Pepsin A, aspartyl protease	At1g09751	104	48429	S (RC 1)
23	Carboxylic ester hydrolase	At2g46930	78	46827	S (RC 1)
24	Unknown protein	At3g08030	83	39326	S (RC 2)
25	Protein binding	At3g20820	54	40464	S (RC 2)
26	Pepsin A, aspartyl protease	At1g09750	70	48429	S (RC 1)
28	β-1,3-glucanase 3	At3g57260	359	37722	S (RC 1)
29	Carboxylic ester hydrolase	At1g29670	539	40417	S (RC 1)
30	Lectin like protein	At3g15356	238	29650	S (RC 1)
31	Lectin like protein	At3g15356	252	29650	S (RC 1)
32	Carbohydrate binding	At5g03350	91	30200	S (RC 2)
33	MERI5b; hydrolase	At4g30270	177	30850	S (RC 1)
34	Chitinase	At2g43590	61	29304	S (RC 1)
35	Peptidylprolyl isomerase (ROC4)	At3g62030	44	28532	C (RC 1)
36	Endopeptidase inhibitor	At1g17860	119	22410	S (RC 2)
37	Hypothetical protein	At1g33640	53	56113	S (RC 2)
38	β-1,3-glucanase 2 (PR2)	At3g57260	140	37373	S (RC 1)
39	Peroxidase, putative, PA2	At5g06720	69	32504	S (RC 2)

*encoded in the chloroplast genome.

Apoplastic washing fluids from mock-infected plants were separated by 2-D-gel electrophoresis, picked, trypsinated and used for determination fragment composition by LC-MS/MS. Identification was achieved by data bank queries as outlined under materials and methods. Spot numbers refer to those shown in Fig. 1. S, C, and M indicate predicted signal peptides for the secretory pathway, chloroplasts or mitochondria, respectively. RC indicates reliability classes for the prediction of localization (1 = high, 5 = low). Peptides are shown in Table S1.

**Table 5:** Proteins significantly affected in the apoplast of *Verticillilum longisporum* VL43-infected compared with that of mock-infected *Arabidopsis thaliana* leaves.

Spot No	Similarity to	Name	Locus	Protein score	Expected MW (Da)	signal	Factor*	*p*
V 1	Serine carboxypeptidase S20 familiy protein	SCP20	At4g12910	214	56238	S (RC 1)	2.4	0.0002
V 2	Germin-like protein (GER3)	GLP3	At5g20630	95	22020	S (RC 1)	3.1	0.0174
V 3	Peroxidase, putative	PRX52	At5g05340	373	34650	S (RC 1)	4.1	0.0004
V 4	Peroxidase 34 (class III peroxidase)	PRX34	At3g49120	54	39440	S (RC 3)	2.3	0.0396
V 5	Peroxidase, putative**	P37	At4g37530	64	36388	S (RC 1)	2.2	0.0491
V 7	α-galactosidase, putative	AGAL2	At5g08370	122	44465	S (RC 1)	2.2	0.0107
27	Lectin like protein/carbohydrate binding	CILLP	At3g16530	287	30547	S (RC 1)	0.5	0.0241

*Factor: protein abundance in samples of VL-treated plants/protein abundance in mock-inoculated plants,

**92% homology to PRX At437520.

Only those spots were analyzed that were reproducibly observed in two independent experiments. In each experiment three biological replicates were analyzed. For further details, see Table 4. Spot identification numbers refer to those shown in Figure 1. Peptides are shown in Table S1.

GO term analysis of the cellular compartmentalization of the identified proteins confirmed that the terms “cell wall” and “apoplast” were highly significantly enriched (Figure S1A). Unexpectedly, the term “plastidic part” was also significantly enriched (Figure S1A). To determine whether the presence of putative plastidic proteins indicated considerable contamination, the relative abundance of these proteins in AWF was compared with their abundances in whole leaf extracts. For this purpose, proteins of leaf extracts were separated by 2D gel electrophoresis and the identities of major proteins were analyzed by ESI-LC/MS (Table S2). Based on their staining intensity, three proteins, which were also detected in AWF, i.e., Rubisco (16.5%), oxygen evolving complex (7.7%), and oxygen evolving enhancer protein (7.0%) were calculated to total 31.2% of the protein in leaves. These proteins contributed 0.88% (Rubisco), 0.32% (oxygen evolving complex) and 0.38% (oxygen evolving enhancer protein) to the total staining intensity in AFWs, corresponding to estimated contamination factors of 0.053, 0.041, and 0.054, respectively. Although these factors indicate that the contamination by plastic compounds may be about 10-fold higher than that estimated for the cytosolic marker MDH (Table 2), it confirms that nonspecific leakage of cellular compounds is low and that AWF is strongly enriched in soluble apoplastic proteins. It should furthermore be mentioned that the localization of – at least some of – the putative plastidic proteins is not entirely clear. For example peptidylprolyl isomerase (ROC4) and the oxygen evolving enhancer protein (OEE33) were assigned to both the apoplast and the chloroplast (http://www.arabidopsis.org/servlets) and thus, may point to dual targeting and perhaps different functions of these proteins in different compartments.

GO term analysis of the category “biological process” of all proteins identified in AWFs revealed significant enrichments of the following functional terms: “proteolysis”, “defense response” and “response to abiotic stimulus” (Figure S1B). VL infection caused significant increases of six of these proteins: three peroxidases (PRX52, PRX34, and P37, the latter named after [23]), one germin-like protein (GLP3), one α-galactosidase (AGAL2), and one serine carboxypeptidase-like protein (SCPL20) and a decrease of one protein, a chitin-inducible lectin-like protein (CILLP, Table 5). According to database analyses in Genevestigator (Figure S2) *CILLP*, *P37*, *PRX52*, *PRX34*, and *GLP3* are strongly responsive to pathogens and *PRX34*, *SCPL20*, *PRX52*, and *AGAL2* to brassinolide/H_3_BO_4_, a treatment that induces formation of xylem elements in cell cultures [24].

### *V. longisporum* induced gene expression and enzyme activities validate proteome analysis but reveal different time courses

**Figure 2:**
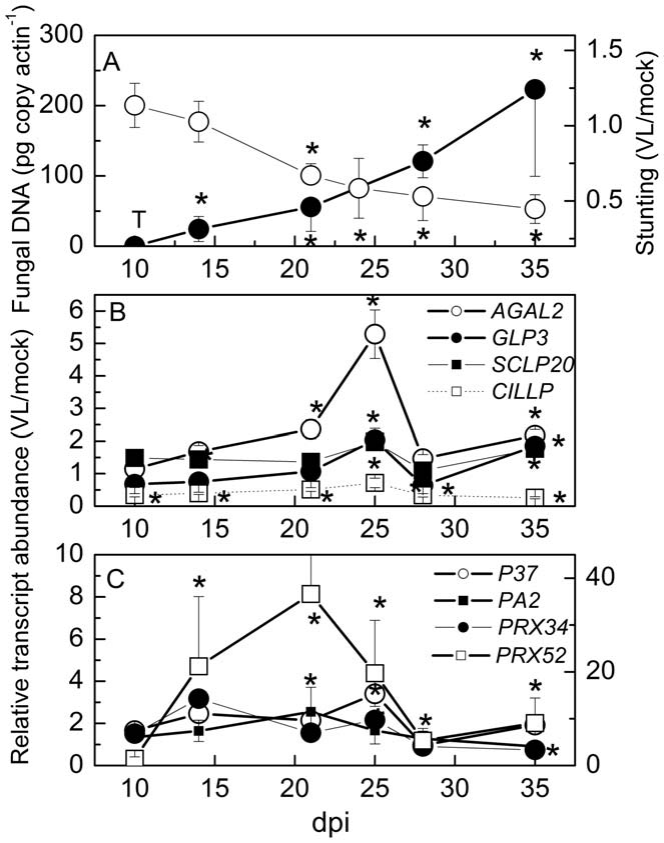
Fungal DNA, stunting of the rosette, and relative transcript abundance of genes encoding apoplastic proteins from *Arabidopsis thaliana* that were either induced or repressed by treatment with *Verticillium longisporum* (VL43). (A) Typical time course of stunting white symbols) and VL43 DNA (black symbols) in Arabidopsis rosettes (n≥4 biological replicates ± SE, except 10 dpi, where pooled samples were analyzed due limited material), T = traces of VL43 DNA detected. Stunting was determined as projected rosette area of VL infected plants/projected rosette area of mock inoculated plants. (B) Transcript levels of *CILLP*, *SCPL20*, *AGAL2* and *GLP3* and (C) transcript levels of the peroxidases *PRX52* (right axis), *PRX34*, *P37* and *PA2* (left axis). Transcript levels were normalized to actin and expressed as transcript abundance in VL-infected plants/transcript abundance in mock-inoculated plants (n≥6 biological replicates ± SE). *indicate significant differences between mock and VL-infected plants.

To obtain independent evidence for the responsiveness of certain AWF proteins to VL, the transcript abundances of the genes for the seven VL-responsive proteins were determined during the time course of VL infection (Fig. 2). Fungal DNA was detected in traces after 10 dpi and increased steadily during the time course of the experiment (Fig. 2A). The increases in fungal DNA corresponded to increasing stunting of the plants (Fig. 2A). VL caused significant decreases in the transcript abundance of *CILLP* at early infection stages (Fig. 2B); *SCLP20* was slightly increased, however, significantly only at 25 and 35 dpi (Fig. 2B). *AGAL2* transcript abundance increased until 25 dpi and decreased afterwards (Fig. 2B). *GLP3* was suppressed during most infection stages, but was elevated at 25 dpi (Fig. 2B).

**Figure 3:**
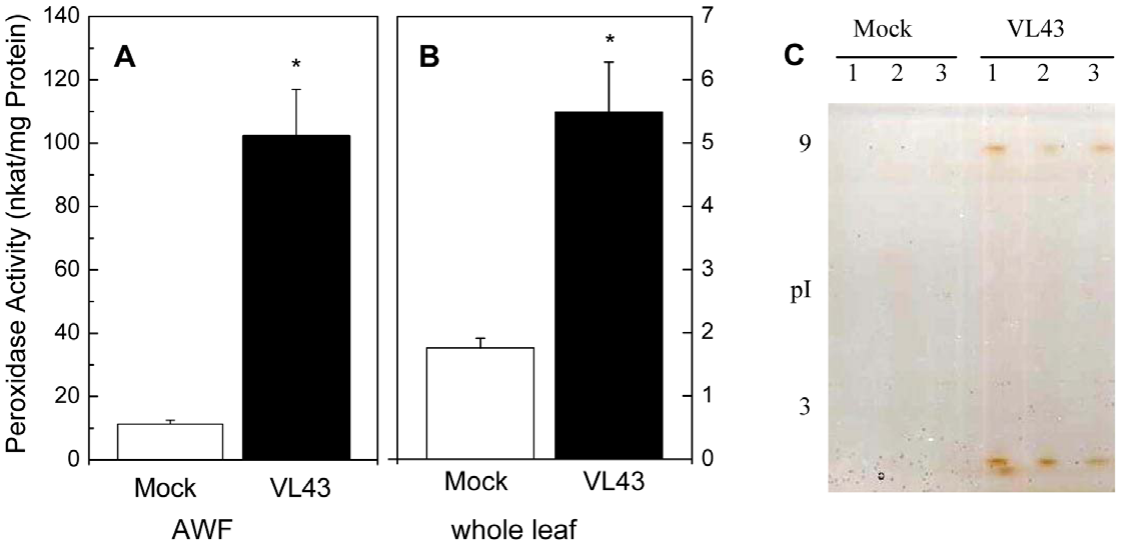
Peroxidase activity in apoplastic (A) and whole leaf extracts (B) and peroxidase activity staining in AWF (C). Data are shown for mock (white columns) and *Verticillium longisporum* (VL43) infected *Arabidopsis* plants (black columns) 25 days post infection. Data indicate means (n = 6, ±SE). *indicate significant differences between mock- and VL-infected plants. For protein separation each lane of an isoelectric focusing gel (pH 3 to 9) was loaded with 3.5 µg AWF protein. Each of the three lanes of mock and VL43-infected plants corresponds to an independent biological replicate of AWF. After isoelectric focusing peroxidase activities were visualized by incubation in guaiacol/H_2_O_2_ solution.

The three VL-responsive PRXs also showed increased transcript abundances after VL infection (Fig. 2C). However, the time courses and degree of stimulation differed. The most profound stimulation was observed for *PRX52* that increased up to 40-fold at 21 dpi. *P37* peaked at 25 dpi, whereas *PRX34* peaked already at 14 dpi and decreased afterwards (Fig. 2C). Increases in PRX after VL infection were also apparent at the level of enzyme activities in both leaf extracts and in AWFs (Fig. 3A,B). The effect was about 10-fold stronger in AWF than in leaf extracts (Fig. 3 A,B) and the induced activities in AWF belonged to both acidic and alkaline PRXs (Fig. 3C).

In addition, the transcript abundance of a non-VL-responsive apoplastic PRX, *PA2* (#39 in Table 4) was measured as negative control. Indeed, the expression of this enzyme was not significantly influenced by VL infection at any time point (Fig. 2C). In conclusion, these data support that VL infection leads to specific increases in six and a decrease in one secreted protein in *Arabidopsis* leaves.

### *V. longisporum* infection affects the apoplastic metabolome and results in cell wall modification of *A. thaliana*

**Figure 4:**
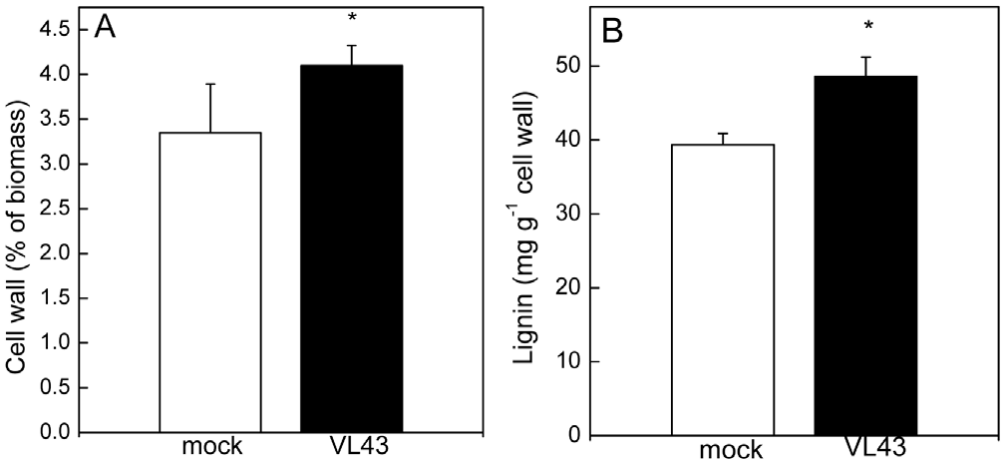
Influence of *Verticillium longisporum* (VL43) on the amount of cell wall material and lignin in mock and VL-infected leaves of *Arabidopsis thaliana*. Data indicate means (n = 9, ±SE). *indicate significant differences between mock and VL-infected plants.

**Figure 5:**
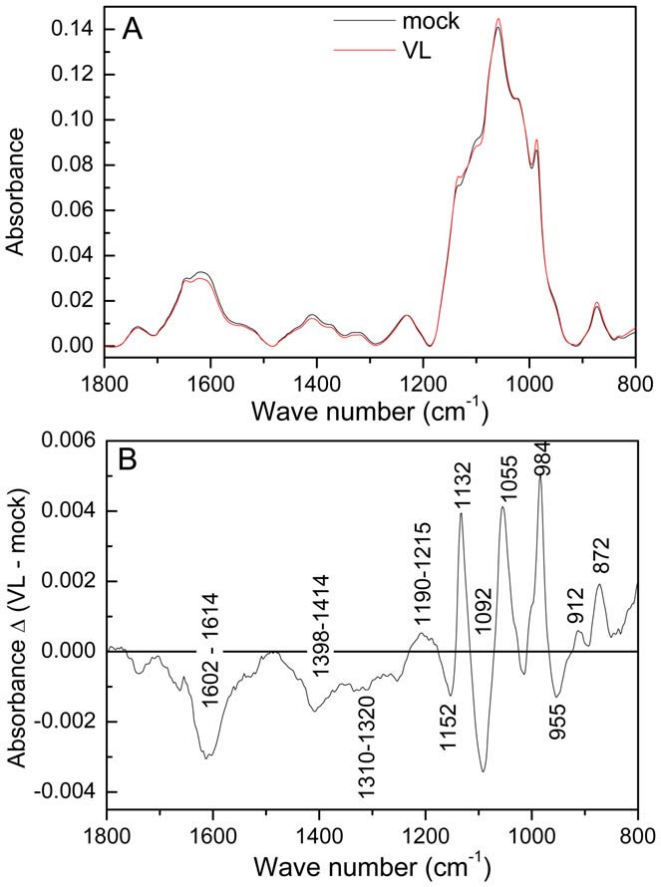
Fourier transform infrared spectra of cell walls of mock- and *Verticillium longisporum* VL43-infected *Arabidopsis thaliana* plants (A) and the difference spectrum (B). The difference spectrum was generated by subtracting the mean spectrum of cell walls from mock infected plants from that of VL-infected plants. Spectra were recorded with a resolution of 4 nm and are shown in the range from 800–1800 cm^−1^; data were baseline corrected and vector normalized. Mean spectra were calculated (n = 8 per treatment) and subtracted.

Since Genevestigator analyses pointed to an influence of VL on cell wall metabolism (Figure S2), cell wall properties of *Arabidopsis* were studied in mock and VL-infected plants. VL infection caused significant increases in the amount of cell wall material and in lignification (Fig. 4A, B). FTIR-ATR spectra were recorded on isolated cell walls to obtain information on their composition in the finger print range from 1800 to 800 cm^−1^ (Fig. 5A). Pronounced differences occurred in the carbohydrate regions between 800 and 1200 cm^−1^ (Fig. 5B). Within this region, cellulose, pectin, rhamnogalacturonan and xyloglucan have overlapping peaks [25]–[27] and therefore, the observed alterations cannot be assigned to distinct compounds. However, cell wall carbohydrate composition was influenced by VL. The region from 1602 to 1614 cm^−1^ has been assigned to esterification of carbonyl groups of pectin [26]. The observed reduction in this area in cell walls from VL-infected plants points to changes in the degree of esterification (Fig. 5B).

**Figure 6:**
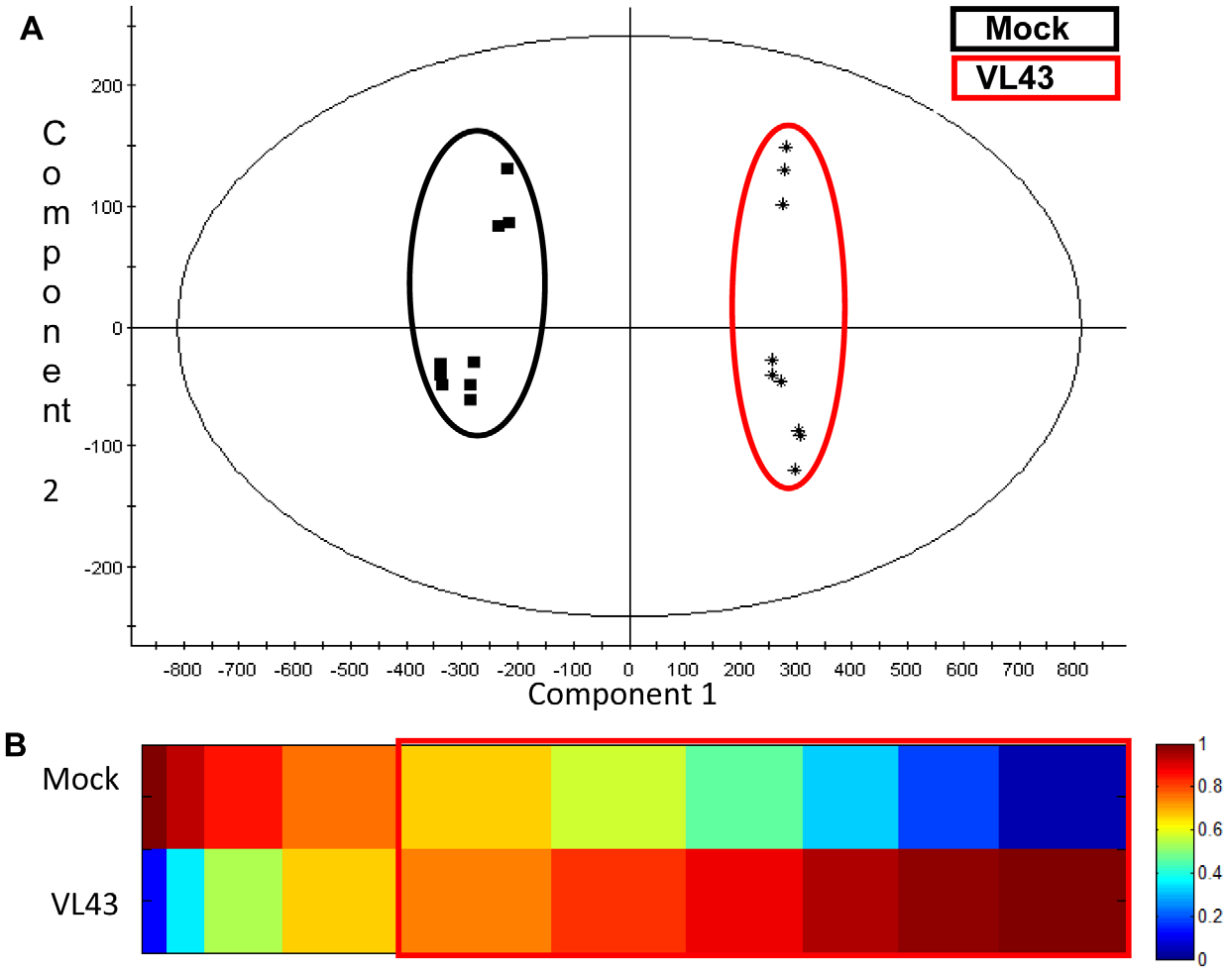
Metabolic fingerprinting of AWFs of mock- and *Verticillium longisporum* VL43-infected *Arabidopsis thaliana* plants. AWF was obtained from *A. thaliana* leaves 21 days post infection and analyzed by UPLC-TOF-MS. (A) PCA plot of compounds in extracted AWFs measured in negative ionization mode. Samples from mock treated plants are framed in black whereas samples from infected plants are framed in red. (B) 1D-SOM matrix after metabolite-based clustering of the 1775 infection markers with *p*<5×10^−4^. Data sets from positive and negative ionisation mode were combined. Red framed prototypes indicate markers with increased intensities after VL infection. The data set includes three biological replicates with three technical replicates per sample. The vertical axis represents the two experimental conditions. The horizontal axis corresponds to the cluster numbers. The intensities were normalized and colour coded according to the indicated scale.

To find out if VL infection also influenced the extracellular metabolome, AWFs of mock- and VL-infected plants were analyzed by metabolite fingerprinting (Table S3). PCA analysis showed a clear separation between the metabolic fingerprints of AWFs from mock- or VL-infected leaves (Fig. 6A). The fingerprinting analysis revealed 1775 marker candidates, which show infection related intensity profiles. Metabolite-based clustering by 1D-SOMs was used to get a global overview of this comprehensive data set (Fig. 6B). The clusters 1–4 include 286 marker candidates (16%) that decreased in AWFs of infected plants. In contrast to that, 1489 marker candidates (84%, cluster 5–10) were enriched in AWFs as consequence of VL infection. It is important to mention that the data still include an unknown number of adduct masses. Although an adduct correction routine has been applied during data preprocessing (see material and methods), the true number of metabolite markers may be less than one-fifth to one-tenth of the calculated numbers. Table 6 shows compounds that were unequivocally identified as infection markers. This list encompasses compounds involved in secondary metabolism (benzoic acid derivates, sinapoyl derivates and lignans), aromatic amino acids (tryptophan, phenylalanine), bioactive fatty acids (9,12,13-trihydroxy-10,15-octadecadienoic acid and 9,12,13-trihydroxy-10-octadecenoic acid), and typical defense related hormones (salicylic acid glycoside, jasmonic acid, Table 6). GC-MS analysis of jasmonic acid (0.42±0.28 nmol g^−1^ f.wt.) and salicylic acid (2.01±0.33 nmol g^−1^ f.wt) in whole leaf extracts did not reveal any significant changes in response to VL at this time point.

**Table 6:** Infection markers identified in leaves of the apoplast of *Arabidopsis thaliana* infected with *Verticillium longisporum* VL43.

Category	Metabolite	Molecular formula	[Da]	RT [min]	SI of Mock (±SE)	SI of VL43 (±SE)	fold-change	*p*	Mode of identification
**Phytohormones**	Jasmonic acid (JA)	C12H18O3	210.1256	5.03	232±17	332±44	1.4	1.13E-05	A, B
	12-Oxo phytodienoic acid (OPDA)	C18H28O3	292.2038	6.57	53±5	101±6	1.9	2.64E-12	A, B
	dinor-OPDA	C16H24O3	264.1725	5.94	71±13	244±37	3.4	5.51E-10	A, B
	Salicylic acid glucoside (SAG)	C13H16O8	300.0845	2.76	287±23	1059±95	3.7	6.77E-14	A, B
	2,5-Dihydroxybenzoic acid glucoside	C13H16O9	316.0794	2.15	4±9	61±25	14.2	8.34E-06	A, B, D
	2,3-Dihydroxybenzoic acid glucoside	C13H16O9	316.0794	2.32	44±34	138±9	3.1	4.75E-07	A, B, D
	2,3-Dihydroxybenzoic acid-3-O-β-D-xyloside	C12H14O8	286.0689	2.52	494±67	1484±137	3	1.47E-12	A
**Oxylipins**	9,12,13-Trihydroxy-10,15-octadecadienoic acid	C18H32O5	328.2251	5.05	157±8	336±14	2.1	3.56E-16	A, B, D
	9,12,13-Trihydroxy-10-octadecanoid acid	C18H34O5	330.2407	5.23	78±7	150±19	1.9	8.01E-09	A, B, D
**Amino acids**	Tryptophan	C11H12N2O2	204.0899	2.5	4286±892	9210±669	2.1	4.82E-10	A, B
	Phenylalanine	C9H11NO2	165.079	1.87	13855±2205	23152±969	1.7	3.44E-09	A, B
**Sinapates**	Sinapoylglucose	C17H22O10	386.1213	3.18	65±10	359±44	5.5	1.38E-12	A, B
	Sinapaldehyde	C11H12O4	208.0736	3.08	78±15	117±13	1.5	2.13E-05	A
**Lignans**	Pinoresinol glucoside	C26H32O11	520.1945	4.2	9±4	79±8	8.5	3.50E-14	A
	Pinoresinol diglucoside	C32H42O16	682.2437	3.56	19±12	142±16	7.4	4.60E-12	A, B, C
	Dehydrodiconiferyl alcohol glucoside (DCG)	C26H32O11	520.1945	3.98	58±8	585±65	10.1	5.19E-14	A, B
**Others**	Raphanusamic acid (RA)	C4H5NO2S2	162.9763	1.5	155±23	256±25	1.7	1.23E-07	A, B

AWF was obtained from *A. thaliana* leaves 21 days post infection and analyzed by UPLC-TOF-MS. Data are means of 3 biological and 3 technical replicates. SI = Signal intensity, Mode of identification refers to A) Exact mass measurement, B) retention time (RT) comparison to authentic standards, C) UV/VIS spectrum, and D) MS/MS fragmentation. *P*-values were calculated by Students t-test.

## Discussion

### Apoplastic proteins in *Arabidopsis* leaves

In the present study approximately 220 apoplastic proteins were detected in *Arabidopsis* leaves, a number in the range of other plant cell wall proteomes [16], [28]. However, it is important to note that protein numbers and composition of cell wall proteomes depend among others on the extraction procedure [16], [28]. Here, soluble proteins and proteins bound by ionic forces were extracted from rosette leaves yielding protein patterns partially overlapping (9 proteins) with those of previous studies applying infiltration and exudation techniques [22], [29], whereas less overlap was found with proteomes from isolated *Arabidopsis* cell walls (2 proteins, [30]) In our study, 35 different, functionally annotated proteins with a secretory signal peptide can be classified according to Jamet et al. [28] into main plant secretome categories as proteins acting on carbohydrates (e.g., glucanase, galactosidase, pectinacetylesterase, chitinase, 25%), proteases (e.g., serine and aspartyl proteases, carboxy peptidases, 18%), oxidoreductases (peroxidases, germin, 21%), proteins with interacting domains (e.g., with other proteins through leucine-rich repeat (LRR) domains, lectins interacting with sugars, or enzyme inhibitors, 21%), lipolytic enzymes (GDSL-like lipase/acylhydrolase, 7%) and unknowns (7%). Some of the interacting proteins identified in our study, MERI5b (At4g30270), LRR domain protein (At3g20820) and ROC4 (At3g62030) are also involved in signal transduction [31], [32]. Other typical signalling proteins such as arabinogalactan proteins and leucine rich receptor kinases, which have been detected in other extracellular proteomes [28], were probably below the detection limit.

Analysis of the *Arabidopsis* xylem sap proteome has not yet been possible. However, the homologs of 30% of the AWF proteins of our study (MERI5b, β-1,3-glucanase, protease inhibitor, curculin-like, chitinase, aspartyl protease, subtilase, PRX34, PRX52, SCPL20, AGAL2) were also present in *Brassica oleracea* xylem sap [10], [33] and the homolog to GLP3 was found in leaf AWF of *Brassica napus* [10]. GO term analysis showed that the AWF was enriched in defence proteins. Indeed, when extracelluar proteins were removed by filtration, strong VL growth found in xylem sap of oilseed rape, whereas addition of these protein to synthetic growth media suppressed fungal proliferation [34]. Xylem saps of soy bean and maize also exhibited antifungal properties [35], [36]. Therefore, an important function of apoplastic proteins is apparently that of a preformed defence to inhibit uncontrolled fungal growth in the extracellular compartment.

### Apoplastic metabolites change in response to *Verticillium longisporum* infection

The plant hormones salicylic acid and jasmonic acid or their derivatives, which are required for systemic acquired resistance and basal pathogen defense [37], were increased in AWF of VL-infected *Arabidopsis*. While genetic analysis of jasmonate-insensitive *Arabidopsis* mutants supports a role of the latter hormone in resistance for *V. dahliae* [38], [39], evidence for a function of salicylic acid in *Verticillium* defense was not obtained [18], [39]. Increases in salicylic acid glycoside, as in our study, were also found in xylem sap of VL-infected oilseed rape [13]. Since the increase in the glycosylated hormone was strictly correlated with increases in VL abundance, a direct antifungal action of this compound is unlikely [13]. Here, AWF of VL-infected plants also contained increased concentrations of other salicylate-related, potentially anti-fungal compounds, i.e., dihydroxy benzoic acid glycosides and xyloside, which may serve as storage form or for stabilization of the bioactive compound [40]. Lignans such as pinoresinol and dehydrodiconiferyl alcohol are produced in response to fungal elicitors and have antifungal properties –at least against human pathogens [41], [42]. Although the AWF proteome contains enzymes that may potentially act on glycosides and xylosides, we found that none of them was increased in response to VL. This suggests that VL may somehow prevent increased deglycosylation, thereby, avoiding enhanced formation of bioactive antifungal compounds. On the other hand we found 9,12,13-trihydroxy-10,15-octadecadienoic acid and 9,12,13-trihydroxy-10-octadecenoic acid which are discussed as antifungal compounds [43].

Interestingly, in AWF the bioactive signaling compound jasmonoyl isoleucine [44] was not affected by VL, but jasmonate. Increases in jasmonate triggered lignin deposition in *Arabidopsis* roots, whose cell wall biosynthesis was impaired by application of the inhibitor of cellulose biosynthesis isoxaben [45]. Furthermore, the glycolsylated form of dehydrodiconiferyl, which was enriched in AWF, has been implicated in stimulation of cell division and was attributed to promote lignin biosynthesis [46], [47]. Several studies including ours report that *Verticillium* diseases result in increased lignification [1]. Recently, changes in the ratio guaiacyl-to-syringyl (G/S) were detected in lignin of *V. dahliae* infected tomato [21]. Our data suggest that this may be regulated by the provision of S-precursors since sinapoyl but not coniferyl derivates increased in AWFs in response to VL and therefore may have not been available for lignin biosynthesis.

### *Verticillium longisporum* affects apoplast defense and cell wall metabolism in *Arabidopsis*

Peroxidases formed the largest group of VL-responsive proteins with three of four different AWF PRX isozymes showing increased abundance. Peroxidases have dual functions in plants: they catalyze formation and the consumption of H_2_O_2_, e.g., during lignin formation. The strongest VL response at both the transcriptional and protein level was found for PRX52. Specific studies on this PRX are lacking but it is activated in response to oxidative stresses and pathogens [48], [49] and therefore, probably belongs to the general defense system of *Arabidopsis*.

Since peroxidases are stable proteins [23], it is not surprising that increased protein abundance can be found in the apoplast, even though transcription was increased only transiently and much earlier after infection as in the case of PRX34. PRX34 plays an important role in the oxidative burst and attenuates spread of biotrophic, necrotrophic and bacterial pathogens [50]. *In vitro*, it catalyzes the copolymerization of S and G-units into lignin [51] and, therefore, may contribute to the increases in lignin observed here. PRX34 also mediates root elongation [52]. In this context, it is interesting to note that VL43 mutant strains, in which a xylem sap-inducible catalase-peroxidase was silenced, were unable to suppress stem elongation of *B. napus* to the same extent as the wildtype strain and it was assumed that the inability to remove reactive oxygen species compromised the infectivity of the pathogen [53].

Lignification is an important barrier preventing the spread of pathogenic fungi. Studies comparing resistant and susceptible cultivars of pepper, cotton, cauliflower, broccoli and tomato provided evidence that peroxidases and increased lignification are involved in the defense against *V. dahliae* and *V. albo-atrum* [21], [54]–[58]. Similarly, Eynck et al. [59] found that cell wall re-inforcement and lignification in the hypocotyl limited the spread of *V. longisporum* in a resistant cultivar of *B. napus*, although the fungus was able to colonize the roots of both cultivars. Our data indicate that these defenses are also activated in VL-infected *Arabidopsis*. However, the velocity and magnitude of defense activation was probably insufficient to prevent colonization with VL eventually. Recently, *Arabidopsis* ecotypes with higher VL resistance have been identified [60]. Future experiments will have to clarify the contribution of PRX to this trait. However, this may be difficult because of the functional redundancy of PRXs in plants [23].

Germins also belong to the plant basal defense system. Germins have been classified in five subfamilies, respond differentially to various stresses, and some have antifungal activities involving H_2_O_2_ formation [61]. For example, QTL analysis in rice identified a germin-like family protein conferring tolerance to a broad spectrum of pathogens [62]. Over-expression of GLP1 from sugar beet in *Arabidopsis* increased H_2_O_2_ production and strongly suppressed root colonization by *Rhizoctonia solani* and *V. longisporum* [63]. Whether GLP3, which was identified as an abundant and VL-induced AWF protein in our study, has similar functions remains to be shown. Previous analyses did not provide evidence that GLP3 results in H_2_O_2_ production [64]. Genevestigator analysis suggests that GLP3 is more strongly induced by abiotic (cold, salt) than by biotic stresses. Mild activation by some pathogens, e.g., *Erysiphe oronti* was found (Figure S2). However, our qRT-PCR data show that the time point of analysis is important since its expression was only transiently increased and was lower than that of non-VL-infected controls during most stages. This observation suggests that interaction with VL may actively suppress potential defenses as observed even more drastically for CILLP both at the transcriptional and protein level. CILLP was previously identified as a chitin-inducible protein [65] and therefore, may be important in plant-fungal interactions.

An interesting result of the Genevestigator analysis was that transcript levels of the VL-induced proteins, AGAL2 and SCPL20, both were strongly activated by brassinolide/H_3_BO_4_ treatment, which triggered formation of tracheary elements in tissue cultures [24]. In our study transcript abundances of both genes increased after VL infection, but that of *AGAL2* much stronger than *SCPL20*. Notably, our study shows that VL-infected *Arabidopsis* produced higher amounts of cell wall material *in planta* than uninfected plants. Indeed, early anatomical studies of *V. albo-atrum* infected hop revealed hyperplastic xylem formation [66]. Formation of the vascular system is regulated by brassinosteroids, whose levels are controlled by BRS1 ( = SCPL24, [67]). However, the transcriptional patterns of *BRS1* and *SCPL20* in AtGeneExpress revealed opposite regulation and indicated that *SCPL20* is increased in senescing leaves (not shown), which was also observed here. Since SCPLs belong to the large, functionally diverse family of serine proteases with more than 200 members in *Arabidopsis* their analysis is difficult [68]. SCLP20 is similar to a wounding- and jasmonate-induced SCPL of tomato [69] and therefore may be involved in defense.

AGAL2 functions in leaf development [70]. Heterozygous T-DNA insertion lines with reduced *AGAL2* levels displayed curly leaves pointing to a role of AGAL2 in cell wall loosening [70]. AGAL2 belongs to a small group of three galactosidases that cleave melibiose into glucose and galactose. The latter compound is an important constituent of hemicelluloses. Deregulation of galactose availability may have resulted in the profound effects of VL on cell wall carbohydrate composition, which have been observed here.

In future studies, it will be important to test if the VL responsive apoplastic proteins identified in this study play functional roles in VL resistance. For this purpose gain- and loss-of-function mutants for these proteins (e.g. T DNA insertion lines and overexpressing lines) will be generated and tested for VL growth and disease symptoms.

### Conclusion

The secretome of soluble and ionically bound proteins in *Arabidopsis thaliana* leaves is enriched in proteins with defense functions. VL infection resulted in specific changes in the apoplastic metabolome and proteome, affecting the abundance of a high number of metabolites and seven extracellular proteins. Six proteins, with overlapping functions in defense, development and cell wall metabolism (three peroxidases, germin, serine carboxypeptidase, α-galactosidase) were significantly increased and one protein with putative functions in plant-fungal interactions (lectin-like protein) was decreased in response to VL infections. VL triggered increased formation of cell wall material with increased lignification in infected plants. FTIR spectra suggested massive modification of cell wall carbohydrates, probably with major changes in the degree of esterification. Transcriptional analyses of the VL-affected proteins revealed different time courses with slow induction of most defense proteins, except PRX52. In contrast, *CILLP* transcript levels were suppressed earlier. It remains to be explored whether the slow induction is due to VL effectors that might delay plant defense responses and facilitate VL colonization of the extracellular compartment. Since some proteins such as AGAL2 and PRX34 have documented functions in cell elongation [52], [70] their induction may have contributed to the observed cell wall modifications and stunting.

## Materials and Methods

### *Arabidopsis* growth conditions and monitoring of *Verticillium* induced damage

*Arabidopsis thaliana* (ecotype Columbia 0) were grown and inoculated with *Verticillium longisporum* as described previously [11]. After germination on agar, *Arabidopsis* seedlings were removed, roots were wounded by cutting the tip, and plants were potted into soil (Typ T25, Fruhstorfer, Vechta, Germany) together with 2×10^7^ spores of *V. longisporum* in 10 ml sterile water applied directly to the roots. Control plants were treated in the same way without spores. The plants were grown at 20°C, 60% relative air humidity, and 120 µmol m^−2^ s^−1^ photosynthetic active radiation (8 h light/16 h dark cycle), watered with tap water and fertilized once a week with Wuxal (Aglucon, Düsseldorf, Germany). Infection symptoms were monitored by measuring the projected leaf area of digital photos (Casio QV R52) with software developed for leaf area measurement (DatInf GmbH, Tübingen, Germany).

For determination of the pigment content 30 mg of frozen leaf powder was thoroughly mixed with 5 ml 80% acetone, incubated for 20 min in darkness on ice, and centrifuged at 4850 *g* (15 min, 4°C). Absorbance of the supernatant was measured for chlorophyll a at 663 nm, chlorophyll b at 646 nm and carotenoids at 470 nm, respectively, and used to calculate pigment concentrations after Lichtenthaler and Wellburn [71].

For measurement of electrolyte leakage twenty-five leaf discs (diameter 8 mm) were punched with a cork borer and placed in a 50 ml tube with 20 ml double deionized water (ddH_2_O). Electrolyte conductivity was determined with a conductometer (LF315; WTW, Weilheim, Germany) after 10 min (L_0_) and 24 h (L_t_). Thereafter samples were boiled for 30 min and then cooled to room temperature before measurement of maximum electrolyte conductivity (L_max_). The electrolyte leakage was calculated as: EL (%) = [(L_t_−L_0_)/L_max_]*100.

The analyses were conducted in three (proteome and physiological analyses) or two (metabolome) independent experiments with plants harvested 21–25 days post inoculation (dpi).

### Fungal culture and quantification of *Verticillium longisporum* DNA

*Verticillium longisporum* isolate VL43 (isolated from *Brassica napus* [4]) was maintained on potato dextrose agar at 4°C. For sporulation, fungal mycelium was transferred to 250 ml liquid potato dextrose medium (Scharlau, Barcelona, Spain) with 0.2 mg ml^−1^ streptomycin sulphate (Sigma, Steinheim, Germany), and incubated at 22°C by horizontal shaking (80 rpm) for 14 d in darkness. The fungal suspension was filtered through a sieve (mesh width 0.5 mm, VWR, Darmstadt, Germany). The filtrate was centrifuged for 10 min at 900 g. The pellet was suspended with ddH_2_O to a spore density of 2×10^6^ conidia per milliliter and used for infection as described above.

Fungal biomass was quantified by determination of fungal DNA in infected plant extracts with real-time PCR. DNA extraction from infected plant leaf material was conducted with the DNeasy Plant Mini Kit (Qiagen, Hilden, Germany). The iCycler System (Bio Rad, Hercules, CA, USA) was used for the amplification and quantification of *Verticillium longisporum* DNA using primers OLG70 (5′-CAGCGAAACGCGATATGTAG-3′) and OLG71 (5′-GGCTTGTAGGGGGTTTAGA-3′). The amplification mix consisted of NH_4_-reaction buffer (16 mm (NH_4_)_2_SO_4_, 67 mm Tris-HCl, 0.01% (v/v) Tween-20, pH 8.8 at 25°C; 3 mm MgCl_2_; 200 µm of each dATP, dTTP, dCTP and dGTP; 0.3 µm of primer OLG70 and OLG71, 0.25 u BIOTaq DNA polymerase (Bioline, Luckenwalde, Germany), 10 nm Fluorescein (BioRad, Hercules, CA, USA), 100,000 times diluted SYBR Green I solution (Invitrogen, Karlsruhe, Germany), 20–30 ng of template DNA and doubly distilled water filled to a total volume of 25 µL. PCR consisted of a 2 min denaturation step at 94°C followed by 36 cycles of 20 s at 94°C, 30 s at 59°C and 40 s at 72°C. The amount of *V. longisporum* DNA was estimated from a calibration curve constructed with purified fungal DNA dissolved in plant matrix. To normalize for different DNA preparations, the Arabidopsis *actin8* gene (At1g49240) was amplified with the primer set act8fow (5′-GGTTTTCCCCAGTGTTGTTG-3′) and act8rew (5′-CTCCATGTCATCCCAGTTGC-3′). The amount of *actin8* DNA in the samples was calculated with a reference plasmid encoding *actin8* sequences. Copy number of the product was calculated from the threshold cycles of duplicate real-time PCR assays using the standard curve.

### Extraction of apoplastic washing fluids

Apoplastic washing fluids (AWF) were extracted using a vacuum infiltration procedure based on the method of Polle et al. [72]. Approximately 10 g of fresh leaves from 10 *Arabidopsis* plants were harvested, washed in de-ionized water and infiltrated for two periods of 5 min at −80 kPa in 250 ml of 100 mM KCl containing 0.005% Triton X-100 for protein extraction or 0.003% Triton X-100 for metabolites. The pressure was slowly released to atmospheric level in the intervening period. The leaves were thoroughly swabbed with filter paper to remove surface humidity, filled into a centrifuge tube (90×25 mm) with a perforated bottom and placed over a 1.5 ml reaction tube in a 50 ml tube. The extracellular washing fluid was collected by centrifugation (8 min, 1000 *g*, 4°C).

### Determination of symplastic contamination

Cytosolic contamination of apoplastic extracts was monitored using the marker enzyme malate dehydrogenase (MDH). For determination of enzymatic activity AWF was filtered through a Sephadex G-25 column (NAP 5 column, Amersham Biosciences, Uppsala, Sweden) to remove low molecular weight compounds.

For MDH activity assay the following reaction mixture was prepared: 500 µl 0.1 M potassium phosphate buffer (pH 7.5), 200 µl H_2_O, 100 µl 1 mM oxalacetic acid and 100 µl AWF or 1∶10 diluted whole leaf extracts [73]. The reaction was started by addition of 100 µl nicotinamide adenine dinucleotidephosphate (NADP), and the change of absorbance at 340 nm was monitored for 5 min at 25°C (Ultraspec 4000, Amersham Pharmacia Biotech, Cambridge, England).

### Leaf extracts, protein quantification and peroxidase activity tests

Whole leaf extracts for enzyme determination were prepared by grinding leaf material in liquid nitrogen and weighing 100 mg of frozen leaf powder into 2 ml of extraction buffer (0.1 M potassium phosphate buffer with 0.5% Triton X-100, pH 7.8 and 100 mg polyvinylpolypyrrolidone). The sample was mixed, centrifuged at 24700 *g* (4°C, 30 min) and the supernatant was filtered through Sephadex G-25 column (NAP 5 column, Amersham Biosciences, Uppsala, Sweden). The protein concentration was quantified by the bicinchoninic acid (BCA) method using the BCA protein quantification kit (Uptima, Montlucon, France) and bovine serum albumin as the standard.

Guaiacol peroxidase activity was measured at 436 nm after Polle et al. [72] The assay contained 50 mM KH_2_PO_4_/K_2_HP0_4_ (pH 5.25), 40 mM guaiacol, 10 mM H_2_O_2_, and 50 µl of three different dilutions of the extract. The increase of absorbance was monitored for 10 min at 25°C (Ultraspec 4000, Amersham Pharmacia Biotech, Cambridge, England). The peroxidase activity was calculated using an extinction coefficient of 25.5 mM^−1^ cm^−1^.

Isoelectric focusing for peroxidase activity staining was performed at 15°C using an electrophoresis system (Phast system, Pharmacia) equipped with minigels providing a pH gradient from pH 3 to pH 9 (Amersham Biosciences, Uppsala, Sweden) as described previously [74]. Aliquots of 3.6 µg of protein were applied per lane. Peroxidase activity was localized by activity staining with 100 µl 30% H_2_O_2_ and 100 µl 98% guaiacol in 50 ml 0.1 M acetate buffer, pH 5.5.

### Two-D gel electrophoresis

Five volumes of a solution consisting of 10% (w/v) TCA and 0.14% β-mercaptoethanol in acetone was mixed with one volume of apoplastic extract or whole leaf extract and stored overnight at −20°C to precipitate proteins. The mixture was centrifuged at 15000 *g* for 15 min at 4°C and then washed three times with 200 µl acetone, 0.14% β-mercaptoethanol. Protein concentration was quantified with a BCA protein quantification kit (Uptima, Montlucon, France). 2-D electrophoresis was performed as described in Görg et al. [75] For 2-D gels precipitated apoplastic proteins (80 µg for spot intensity analysis and 250 µg for spot picking) were dissolved in 350 µl IEF sample buffer (8 M urea, 0.5% 3-[(3-cholamidopropyl)dimethylammonio]propanesulfonic acid [CHAPS], 15 mM dithiothreitol [DTT], 0.5% v/v Pharmalyte 3–10, 0.2% v/v Bromophenol Blue) and loaded on 18 cm IPG strips, pH 3–10 (Amersham Biosciences, Uppsala, Sweden) in ceramic strip holders. After rehydration of the strips for 12 h at 20 V, isoelectric focusing was performed in an Ettan IPGphor Unit (Amersham Biosciences, Uppsala, Sweden) under the following conditions: 2 h at 150 V, 1 h at 200 V, 1 h at 500 V, constant 1000 V for 1000 Vh, followed by a gradient to 8000 V and finally focusing for 28 000 Vh. Focused samples were stored at −20°C.

Prior to loading on to the second dimension gel strips were equilibrated twice for 15 min in 10 ml equilibration buffer (6 M urea, 30% (w/v) glycerol, 2% sodium dodecyl sulfate [SDS]) containing 10 mM DTT in the first step and 100 mM iodoacetamide in the second step, respectively. For the second dimension equilibrated gel strips were placed on top of vertical 12% SDS polyacrylamide gels and covered with 1 ml 0.5% hot low-melting point agarose in electrode buffer (25 mM 2-amino-2-hydroxymethyl-propane-1,3-diol [Tris], 192 mM glycine, 0.1% SDS and 0.03% Bromophenol Blue). Six gels were simultaneously run for 30 min at 600 V, 400 mA, 13 W and approximately 4 h at 3000 V, 400 mA, 100 W at 10°C in an Ettan DALTsix unit (Amersham Biosciences, Uppsala, Sweden) until the Bromophenol Blue front reached 1.5 cm before the end of the gel.

### Protein staining and statistical analyses

For analysis of spot intensities gels were silver stained [76]. Gels were incubated overnight in 500 ml fixing solution (50% v/v ethanol, 12% v/v acetic acid, 0.05% v/v 37% formaldehyde), followed by three washing steps with 30% ethanol for 20 min each. After the washing step gels were impregnated for 1 min in 0.01% w/v sodium thiosulphate, washed three times 20 s with pure water and stained 20 min in 0.1% w/v silver nitrate, 0.075% v/v formaldehyde (37%). Following two washes in pure water proteins were visualized by incubation in revealing solution (3% w/v sodium carbonate, 0.0002% w/v sodium thiosulphate, 0.05% v/v 37% formaldehyde). The staining procedure was stopped by washing in 50 mM ethylenediaminetetraacetic acid (EDTA) for 1 h once a suitable intensity had been reached. Gels were scanned using Fluor-S Multiimager (Bio-Rad, Dreieich, Germany) as TIFF-files.

To compare protein patterns of VL-treated and non-infected plants six gels per treatment (three independent biological replicates, two technical replicates) were monitored. Gels belonging to one treatment were analyzed as a group by Proteomweaver software (Version 3.1.07, Definiens, München, Germany). Protein spots were identified automatically, adjusted manually where necessary and merged by Pair Matching or Multi Matching. The grouped gels were normalized using standard settings of the program and used for statistical analyses of staining intensities of matching spots by Student's t-test. *P*≤0.05 was considered to indicate significant differences.

### Protein identification and mass spectrometry

Gels for spot picking were stained overnight with Coomassie Brilliant Blue (0.1% w/v CBB G-250, 1.7% w/v phosphoric acid, 10% w/v ammonium sulfate, 20% v/v methanol) as described by Neuhoff et al. [77]. Gels were then destained by repeated washing steps with ddH_2_O until the background of the gels was clear.

Spots were excised from Coomassie-stained 2 D gels followed by in-gel trypsin digestion [78]. Prior to digestion gel pieces were washed twice for 15 min with ddH_2_O, twice for 15 min with 50% methanol and finally dehydrated for 20 min with 200 µl acetonitrile in 0.5 ml reaction tubes. After drying, gel pieces were covered with 20 µl trypsin (Promega, Madison, USA), incubated for 1 h at 4°C and subjected to protein digestion for 30 min at 58°C. The reaction was stopped and peptides extracted with 200 µl 5% v/v formic acid for 30 min. The supernatant was transferred into new tube and gel pieces were extracted again with 200 µl 50% v/v acetonitrile, 5% v/v formic acid for 30 min. The combined supernatants were concentrated to dryness in a vacuum concentrator (SPD speed vac, Thermo Savant, Holbrook, USA). For mass spectrometry peptides were dissolved in 5 µl of 0.1% v/v formic acid. Peptides were analyzed by electro spray ionization liquid chromatography/mass spectrometry (ESI-LC/MS) (Esquire 3000, Bruker Daltonik, Bremen, Germany) and identified by searching the NCBInr database using Mascot software (Matrix Science, London, UK). Settings for peptide searches were: ion charge, 2+ and 3+, monoisotopic ions, carbamidomethyl-C as fixed modification, oxidation-M as variable modification, missed cleavage 1, peptide tolerance 1.4 Da, MS/MS tolerance 0.4 Da, standard scoring using significance threshold p<0.05. Identified proteins were analyzed with TargetP (version 1.1) to predict their subcellular localisation with a certain reliability class (RC) [79]. Small values indicate high reliability.

### Quantitative real time polymerase chain reaction (qRT PCR)

Leaf samples harvested weekly between 7 and 35 dpi were used for RNA extraction after Chang et al. [80]. DNA in the extracts was removed with DNase (Turbo DNase buffer and Turbo DNase, Ambion, Austion, USA) and first strand cDNA was synthesized from 5 µg of RNA using reverse transcriptase and oligo dT primers (First strand cDNAkit, MBI Fermentas, St Leon-Roth, Germany) according to the manufacturer's recommendations. Gene-specific primers (Table S4) were designed by using the Primer3 software (http://frodo.wi.mit.edu/primer3/) [81] and were obtained from MWG (Ebersberg, Germany). The relative transcript abundance was detected in an iCycler using iQ SYBR Green Supermix (Bio-Rad). Actin was used for normalization. The relative expression ratio was analyzed using the relative expression software tool (http://www.gene-quantification.de/) [82].

### Extraction of cell walls and lignin determination

For extraction of cell walls 200 mg frozen leaf powder was extracted by mixing for 30 min in 1.5 ml 100 mM potassium phosphate buffer, 0.5% Triton X-100 (pH 7.8). The mixture was centrifuged (10 min, 14000 *g*), the supernatant was discarded and the pellet was washed again with 1.5 ml 100 mM potassium phosphate buffer, 0.5% Triton X-100 (pH 7.8), followed by a washing step with 1.5 ml methanol. The obtained cell wall material was dried at 70°C.

Lignin was determined by the acetylbromide method as described by Brinkmann et al. [83] Aliquots of 0.5, 1, and 2 mg of cell wall material were mixed with 250 µl of 25% v/v acetylbromide in acetic acid and incubated for 30 min at 70°C. Samples were rapidly cooled on ice, mixed with 250 µl of 2 M NaOH, and centrifuged for 5 min at 16000× g. 139 µl supernatant were mixed with 2.8 µl hydroxylamine solution and 1.25 ml acetic acid. The absorbance was measured at 280 nm (Beckman DU 640, Beckman Coulter GmbH, Krefeld, Germany). Calibration curves were generated with increasing amounts of 18–270 µg of commercial lignin (Sigma, Steinheim, Germany). The extinction coefficient of alkaline spruce lignin was ε = 8.41 g^−1^ cm^−1^.

### Fourier transform infrared (FTIR) spectroscopy

FTIR analysis was performed with an FTIR spectrometer (Equinox 55, Bruker Optics, Ettlingen, Germany) with an attached unit to measure attenuated total reflectance (ATR unit) as described previously [84], [85]. FTIR spectra were recorded for the wave number range of 4000 to 800 cm^−1^ with a spectral resolution of 4 cm^−1^. To improve the signal-to-noise ratio 16 spectra per sample were added and averaged. FTIR data were evaluated using the OPUS version 5.0 software (Bruker). Spectra were vector normalized and the first derivative was used to compare differences in the spectra. Eight individual plants were analyzed per treatment and data were reported as mean spectra.

### Metabolite fingerprinting analysis

Aliquots of 200 µl AWF were extracted with 150 µl methanol and 500 µl methyl-*tert*-butylether (MTBE) adapting a protocol of Matyash et al. [86]. After shaking for 1 h in the dark 120 µl water were added. The samples were incubated for 10 min and centrifuged for 15 min at 1500 rpm and 20°C to allow phase separation. The polar and non-polar phases were transferred carefully to a new vial avoiding contamination with material of interphase and pellet. The combined phases were evaporated under a nitrogen stream and resolved in 50 µl acetonitrile/methanol/water (1∶1∶12, *v/v/v*).

The metabolite fingerprinting analysis was performed by Ultra Performance Liquid Chromatography (UPLC, ACQUITY UPLC System, Waters Corporation, Milford, USA) coupled with a photo diode array detector and an orthogonal time-of-flight mass spectrometer (TOF-MS, LCT Premier, Waters Corporation, Milford, USA). For analysis 2 µl of the sample were injected and separated on an ACQUITY UPLC HSS T3 column (1.7 µm, 1×100 mm, Waters Corporation, Milford, USA) with a flow rate of 0.2 ml min^−1^ at 40°C. Mobile phases A and B were water and acetonitrile with 0.1% formic acid, respectively. The gradient program was set up as follows: 0–0.5 min 1% solvent B, 0.5–3 min from 1% to 20% solvent B, 3–8 min from 20% to 95% solvent B, 8–10 min 95% solvent B, 10–14 min equilibration with 1% solvent B.

The TOF-MS was operated in positive and negative electrospray ionisation (ESI) mode as described by Nahlik et al. [87].

Data processing of the raw spectral data were performed by the MarkerLynx Application Manager for MassLynx 4.1 software (Waters Corporation, Milford, USA) resulting in two data matrices, one for all samples of the positive ionization mode and one for the negative ionization mode. Data deconvolution, peak integration and alignment were performed over the retention time range from 0.3 to 10 min and the mass range from 50 to 1200 Da with the following parameters: peak width at 5% height: 12 sec; peak-to-peak baseline noise: 200 cps; no smoothing applied; intensity threshold: 50 counts (negative ionization) and 100 counts (positive ionization); mass window: 0.03 Da; retention time window: 0.2 min; noise elimination level: 5. Isotopomers were removed. The resulting data matrix was exported as csv-file. Principle component analysis (PCA) was conducted with MarkerLynx Extended Statistics.

For further data processing like ranking and filtering of the data, adduct identification and correction of the raw masses, combining of the data matrices as well as for clustering and visualization the toolbox MarVis (MarkerVisualization, http://marvis.gobics.de) has been used. This toolbox includes the routines MarVis Filter and MarVis Cluster [88]. Datasets were imported into MarVis Filter as csv-file and a Kruskal-Wallis test was performed to extract high quality marker candidates with a p-value<5×10^−4^. Adducts were corrected according to the following rules: positive ionization: [M+H]^+^, [M+Na]^+^, [M+NH_4_]^+^; negative ionization: [M-H]^−^, [M+CH_2_O_2_-H]^−^, [M+CH_2_O_2_+Na-2H]^−^. Subsequently positive and negative datasets from one experiment were combined.

For detection of infection markers within the dataset the tool MarVis Cluster were used. Data were clustered and visualized by means of one-dimensional self-organizing-maps (1D-SOMs). During normalization sample aggregation is performed on mean values and marker scaling by the Euclidean norm (2-norm). Clusters with intensity pattern indicating enhanced levels after infection were selected. Marker candidates represented by these clusters were putatively identified by automated database search within a mass deviation of 5 mDa: AraCyc (http://www.arabidopsis.org), MetaCyc (http://metacyc.org), KEGG (http://www.genome.jp/kegg), Knapsack (http://kanaya.naist.jp/KNApSAcK) and LIPIDMAPS (http://www.lipidmaps.org). The identity of the infection markers was confirmed by further methods as MS^2^ analysis, UV/VIS analysis or comparison of retention time with authentic standards.

The identity of 9,12,13-trihydroxy-10,15-octadecadienoic acid and 9,12,13-trihydroxy-10-octadecenoic acid was confirmed by MS^2^ analysis. For that samples were analyzed by LC 1290 Infinity (Agilent Technologies, Santa Clara, USA) coupled with an 6540 UHD Accurate-Mass Q-TOF LC/MS instrument with Agilent Jet Stream Technology as ESI source (Agilent Technologies, Santa Clara, USA). For LC a ZORBAX RRHD Eclipse Plus C18 column (2.1×150 mm, 1.8 µm particle size, Agilent Technologies, Santa Clara, USA) was used at a temperature of 40°C, a flow rate of 0.4 ml/min and with the solvent system and a comparable gradient as applied for UPLC analysis.

The Q-TOF MS instrument was operated with a detection frequency of 4 GHz in the targeted MS^2^ mode. The source conditions were: gas temperature: 300°C; drying gas flow: 8 L min^−1^; nebulizer pressure: 35 psi; sheat gas temperature: 350°C; sheat gas flow: 11 L min^−1^; VCap voltage: 3.5 kV; nozzle voltage: 100 V; fragmentor voltage: 175 V. All samples were ionized in negative ionization mode and determined in targeted MS^2^ mode with collision energy of 25 eV. Isolation of precursor ions occurred within the narrow isolation width of ∼1.3 m/z. For exact mass measurement the reference mass correction with trifluoroacetic acid ([M-H]^−^ 112.98559) and HP-921 = hexakis-(1H,1H,3H-tetrafluoro-pentoxy) phosphazene ([M+HCO_2_]^−^ 966.00073) as reference compounds were used. The LC/MS was operated under Mass Hunter B03.01 (Agilent Technologies, Santa Clara, USA). Data were acquired by Mass Hunter Workstation Acquisition software B.03.01 service pack 3 (Agilent Technologies, Santa Clara, USA). Mass Hunter Qualitative Analysis B.03.01 service pack 3 (Agilent Technologies, Santa Clara, USA) was used for data analysis. The MS^2^ spectra of 9,12,13-Trihydroxy-10,15-octadecadienoic acid and 9,12,13-Trihydroxy-10-octadecenoic acid were compared with the fragment information of the authentic standard.

Salicylic acid and jasmonic acid in leaf extracts were measured by GC/MS as described before [89].

### Statistical and bioinformatic analyses

Data are means of n = 5 to 8 biological replicates (± SD). Statistical analyses were performed using the program Statgraph (Centurion XV, St. Louis MO) employing Student's t-test, analysis of variance followed by a multiple range test or by a non parametric test (Mann-Whitney), when data were not normal distributed. Values of *p*≤0.05 were considered to indicate significant differences.

Bioinformatic analyses were performed with open source software. Lists with identified Arabidopsis gene loci according to The Arabidopsis Information Resource (TAIR, http://arabidopsis.org/) were analyzed and visualized with the standard settings in AgriGO (http://bioinfo.cau.edu.cn/agriGO/) to obtain information on significantly enriched gene ontology (GO) terms [90]. The list with the AGI loci of the proteins that were significantly affected by VL were furthermore subjected to *in silico* analyses using Genevestigator (https://www.genevestigator.com/) [91].

## Supporting Information

Figure S1 **GO term enrichment analysis of apoplastic proteins for the categories “cellular compartment” (A) and “biological process” (B).**
(XLS)

Figure S2 **Data base microarray analysis of transcriptional pattern of** ***V. longisporum*** **affected proteins in response to stress treatments.**
(XLS)

Table S1 **Peptide information on the identified apoplast proteins of** ***Arabidopsis thaliana*** **rosette leaves.**
(XLS)

Table S2 **Proteome analysis of major proteins in whole leaf extracts of** ***Arabidopsis thaliana*** **.**
(XLS)

Table S3 **Original metabolome data set.**
(XLS)

Table S4 **Primers used for verification of expression changes of VL-responsive proteins.**
(XLS)
